# Fibro–Adipogenic Progenitors Cross-Talk in Skeletal Muscle: The Social Network

**DOI:** 10.3389/fphys.2019.01074

**Published:** 2019-08-21

**Authors:** Beatrice Biferali, Daisy Proietti, Chiara Mozzetta, Luca Madaro

**Affiliations:** ^1^Department of Biology and Biotechnology “C. Darwin,” Sapienza University of Rome, Rome, Italy; ^2^Institute of Molecular Biology and Pathology (IBPM), CNR National Research Council of Italy, c/o Department of Biology and Biotechnology “C. Darwin,” Sapienza University of Rome, Rome, Italy; ^3^IRCCS Santa Lucia Foundation, Rome, Italy; ^4^DAHFMO-Unit of Histology and Medical Embryology, Sapienza University of Rome, Rome, Italy

**Keywords:** FAPs, fibrosis, cytokine – immunological terms, muscle regeneration, stem cell

## Abstract

Skeletal muscle is composed of a large and heterogeneous assortment of cell populations that interact with each other to maintain muscle homeostasis and orchestrate regeneration. Although satellite cells (SCs) – which are muscle stem cells – are the protagonists of functional muscle repair following damage, several other cells such as inflammatory, vascular, and mesenchymal cells coordinate muscle regeneration in a finely tuned process. Fibro–adipogenic progenitors (FAPs) are a muscle interstitial mesenchymal cell population, which supports SCs differentiation during tissue regeneration. During the first days following muscle injury FAPs undergo massive expansion, which is followed by their macrophage-mediated clearance and the re-establishment of their steady-state pool. It is during this critical time window that FAPs, together with the other cellular components of the muscle stem cell niche, establish a dynamic network of interactions that culminate in muscle repair. A number of different molecules have been recently identified as important mediators of this cross-talk, and its alteration has been associated with different muscle pathologies. In this review, we will focus on the soluble factors that regulate FAPs activity, highlighting their roles in orchestrating the inter-cellular interactions between FAPs and the other cell populations that participate in muscle regeneration.

## Introduction

Skeletal muscle is the most abundant tissue in healthy humans, accounting for 40% of body weight. It is composed of multinucleated contractile cells called myofibers, which are formed during development by fusion of differentiated mononuclear muscle cells, and their number remains constant during post-natal growth. The regenerative potential of skeletal muscle relies primarily on satellite cells (SCs), the prototypical muscle stem cells. Upon muscle injury SCs enter the cell cycle, proliferate, and differentiate to repair damaged myofibers, while self-renewing to repopulate the reserve pool (Feige et al., 2018).

Recently, several studies have indicated that the establishment of functional cross-talk between SCs and other cell types within the muscle niche, including motor neurons, endothelial cells, immune cells, fibrogenic cells, and adipogenic precursors, is crucial for muscle repair and homeostasis (Tatsumi et al., 2009; Joe et al., 2010; Uezumi et al., 2010; Heredia et al., 2013; Saccone et al., 2014; Kuswanto et al., 2016; Verma et al., 2018; Giordani et al., 2019; Lukjanenko et al., 2019). Indeed, different stem/progenitor cell types are recruited to the regenerative niche and influence muscle regeneration either by directly differentiating into muscle cells or by releasing paracrine factors (i.e., growth factors, cytokines) that control the regenerative response of SCs (Pannérec et al., 2012). Among the non-cellular components of the SCs niche the extra-cellular matrix (ECM) plays a crucial role by undergoing a transient remodeling during acute injury, followed by a prompt termination to avoid pathological fibrosis and tissue degeneration. Although recent findings have shown that myogenic cells can produce ECM components (Fry et al., 2017b; González et al., 2017; Baghdadi et al., 2018), and a recently identified population of interstitial tenocytes has been implicated in ECM deposition *in vivo* (Giordani et al., 2019), the main cellular sources of ECM proteins are fibroblasts, myo-fibroblast, and fibro–adipogenic progenitors (FAPs) (Serrano and Muñoz-Cánoves, 2010; Lemos et al., 2015; Contreras et al., 2016; Mueller et al., 2016).

Since their discovery FAPs have attracted a considerable attention (Joe et al., 2010; Uezumi et al., 2010), in particular, their phenotypical plasticity, which appears critical for efficient muscle repair. FAPs have been defined as multi-potent progenitors, having the ability to differentiate into fibroblasts, adipocytes, and possibly into osteoblasts and chondrocytes, although not into myoblasts (Joe et al., 2010; Uezumi et al., 2010). They share the expression of cell surface markers, such as Sca-1 and PDGFRα with mesenchymal stem cells and can therefore be broadly defined as mesenchymal precursors (Joe et al., 2010; Mueller et al., 2016; Judson et al., 2017; Malecova et al., 2018; Giordani et al., 2019).

Under quiescent conditions FAPs frequently localize close to blood vessels but unlike pericytes FAPs reside outside the capillary basement membrane and do not express NG2 (Joe et al., 2010).

However, upon muscle injury, FAPs become activated, proliferate and expand, and provide a transient favorable environment to promote SCs-mediated regeneration (Joe et al., 2010; Heredia et al., 2013; Mozzetta et al., 2013). FAPs expansion is critical during regeneration in order to sustain SCs differentiation in a paracrine manner and to maintain the SCs pool (Wosczyna et al., 2019). Indeed, *in vivo* depletion of FAPs clearly established their absolute requirement for regeneration and long-term maintenance of skeletal muscle (Wosczyna et al., 2019). However, as regeneration proceeds, FAPs are cleared from the regenerative niche by apoptosis (Lemos et al., 2015) and failure in doing so has been associated with their pathological accumulation and with a number of muscle dysfunctions. In fact, beyond their supportive role in muscle regeneration, FAPs have been identified as the major source of infiltrating fibroblasts and adipocytes in degenerating dystrophic muscles (Uezumi et al., 2010, 2011; Mozzetta et al., 2013; Kopinke et al., 2017). Similarly, in chronic atrophic conditions, caused by moto-neurons deficits, increased fibrosis is associated with accumulation of FAPs in the interstitium of denervated muscles (Contreras et al., 2016; Fry et al., 2017a; Madaro et al., 2018; Rebolledo et al., 2019). Likewise, intra-muscular fatty infiltration and obesity-associated muscle dysfunctions have been also linked to FAPs accumulation and fibro–adipogenic differentiation (Dammone et al., 2018; Gorski et al., 2018; Kang et al., 2018; Pagano et al., 2018; Buras et al., 2019).

These findings emphasize that the FAPs lineage decisions are dramatically influenced by signals released in their microenvironment, whose pathological alteration might culminate in excessive ECM accumulation (Lemos et al., 2015; Contreras et al., 2016; Dammone et al., 2018; Madaro et al., 2018), acquisition of altered cell fates, as in the case of heterotypic ossification (Lees-Shepard et al., 2018), and impaired myogenesis. In physiological conditions, FAPs’ cross-talk with other cell populations is emerging as an important and finely orchestrated process crucial for a successful muscle regeneration. While it is now well established that a cross-talk between SCs and fibrogenic cells is necessary for efficient SCs expansion in response to injury, and to prevent interstitial fibrosis accumulation (Murphy et al., 2011; Fry et al., 2017b; Lukjanenko et al., 2019), increasing evidence indicates that FAPs also actively interact with immune cells in a finely tuned manner (Heredia et al., 2013; Lemos et al., 2015; Malecova et al., 2018; Moratal et al., 2018).

Taken together, these observations demonstrate that FAPs orchestrate a plethora of processes involved in regenerative myogenesis, which have been recently reviewed elsewhere (Wosczyna and Rando, 2018). In this mini-review, we will instead specifically focus on the secreted signals, cytokines, and paracrine factors controlling FAPs function and those released by FAPs monitoring the different cell types involved in muscle repair. We will first describe the signals secreted by the various cell populations present in the regenerative niche known to directly influence FAPs activity and then discuss the signals released by FAPs themselves, highlighting their cellular targets and functions (Table 1).

**TABLE 1:** Schematic table illustrating the principal molecules, the producing and target cells, and the corresponding biological effects, that have been shown to act on, or be released by, FAPs.

**FAPs social network**					

**Molecule**	**Production cells**	**Target**	**Effects**	**References**	**Experimental conditions**
TGF-β	Macrophages	FAPs	TGF-β induces FAPs fibrogenic differentiation and blocks TNF-induced FAPs apoptosis.	Uezumi et al., 2011; Lemos et al., 2015; Davies et al., 2016	*In vitro* and *in vivo*
IL-15	Muscle fibers	FAPs	IL-15 stimulates FAPs proliferation and prevents adipogenic differentiation *in vitro* and *in vivo* and promotes FAPs-induced SC differentiation.	Kang et al., 2018	*In vitro* and *in vivo*
IL-4	Eosinophils	FAPs	IL-4 inhibits adipogenic differentiation of FAPs and increases FAPs ability to remove cellular debris enhancing regeneration.	Heredia et al., 2013; Dong et al., 2014	*In vitro* and *in vivo*
IL-13		FAPs	IL-13 promotes FAPs proliferation that supports myogenesis, while inhibits FAPs differentiation into adipocytes.	Heredia et al., 2013	*In vitro* and *in vivo*
TNF-α	Macrophages	FAPs	TNF-α induces FAPs apoptosis preventing excessive deposition of extracellular matrix during regeneration.	Lemos et al., 2015	*In vitro* and *in vivo*
IL-6	FAPs	Myotubes	IL-6 promotes myogenic differentiation.	Joe et al., 2010	*In vitro*
		FAPs	IL-6 promotes pro-atrophic FAPs phenotype during denervation.	Madaro et al., 2018	*In vitro* and *in vivo*
IL-33	FAPs	Regulatory T cells	IL-33 increases Treg cells proliferation promoting muscle repair.	Kuswanto et al., 2016	*In vitro* and *in vivo*
Follistatin	FAPs	Satellite cells	FAPs-secreted follistatin promotes multinucleated myotubes formation.	Mozzetta et al., 2013	*In vitro*
IL-10	FAPs	Myotubes	IL-10 is upregulated in FAPs during muscle regeneration. Its role is still unknown but the hypothesis is that the secretion of IL-10 facilitates myoblast differentiation by preventing the antimyogenic activity of TNF and IL-1β.	Lemos et al., 2012	n/a
BMP1-MMP14	FAPs	Macrophages	FAPs-secreted BMP1 and MMP14 activate TGF-β produced by macrophages in fibrotic DMD muscle.	Juban et al., 2018	*In vitro*
WISP1	FAPs	Satellite cells	FAPs-secreted WISP1 regulates satellite cell expansion and asymmetric differentiation. FAPs-derived WISP1 is lost during aging impairing muscle regeneration.	Lukjanenko et al., 2019	*In vitro* and *in vivo*

*References relative to the evidence described are shown together with the experimental conditions (*in vivo* or *in vitro*) used to identify the described mechanisms. Where experimental evidence is lacking we indicated the experimental procedure with n/a.*

## The Secretome That Regulates FAP*s* Activities

### IL-4 and IL-13 Family

Interleukin-4 (IL-4) and IL-13 are Th2 cytokines, which have been implicated as mediators in the cross-talk between the immune system and FAPs (Heredia et al., 2013). The innate immune system is activated rapidly upon muscle injury and triggers the recruitment of Th2 lymphocytes, macrophages, mast cells, and eosinophils to the injured sites (Tidball and Villalta, 2010; Heredia et al., 2013).

Interleukin-4/IL-13 signaling is crucial for skeletal muscle repair, as demonstrated by studies showing a complete absence of regenerated myofibers, persistence of cellular debris, and an inflammatory infiltrate, in the muscles of *IL-4/IL-13*^–/–^ mice following cardiotoxin-induced injury (Heredia et al., 2013). Although activation of type 2 immune responses has been classically associated with alternatively activated (M2) macrophages (Allen and Wynn, 2011; Palm et al., 2012), eosinophils have been recently identified as the dominant cell source of IL-4 and IL-13 (Heredia et al., 2013) during skeletal muscle regeneration. Specifically, it has been shown that eosinophils secrete IL-4 to activate the regenerative actions of FAPs. Indeed, Heredia et al. (2013) identified FAPs as the cells specifically expressing the IL-4Rα, demonstrating, both *in vitro* and *in vivo*, that FAPs are the cellular targets of IL-4/IL-13 signaling during muscle regeneration. Intriguingly, they also unveiled a previously unrecognized function of FAPs: their capacity to phagocytoze necrotic debris, a crucial process for successful completion of muscle repair (Heredia et al., 2013). In addition, IL-4/IL-13 signaling, via activation of STAT6, promotes FAPs proliferation to support myogenesis, while inhibiting their differentiation into adipocytes (Heredia et al., 2013). Accordingly, in the injured muscles of IL-4-knockout mice, the levels of adipocytes are increased, while *in vitro* and *in vivo* administration of IL-4 inhibits FAPs adipogenesis (Heredia et al., 2013; Dong et al., 2014). In agreement with these observations, glucocorticoids (GCs)-induced repression of IL-4 leads to intramuscular adipogenic accumulation by promoting FAPs proliferation and differentiation into adipocytes (Dong et al., 2014). Since GCs are known to suppress eosinophils, it is likely that they inhibit IL-4 signaling by reducing the number of infiltrating eosinophils upon muscle injury (Dong et al., 2014). Yet, IL-4-polarized, anti-inflammatory macrophages have been shown to induce adipogenesis of human FAPs isolated from dystrophic muscles (Moratal et al., 2018), suggesting that IL-4 signaling might govern more complex cellular interactions than previously expected.

### IL-15

Interleukin-15 is expressed in human skeletal muscle and it has been identified as an anabolic factor involved in muscle growth (Quinn et al., 2002; Furmanczyk and Quinn, 2003). Indeed, IL-15 can decrease protein degradation in muscle (Busquets et al., 2005) and modulate muscle–adipose tissues interactions (Quinn et al., 2005). A recent work identified IL-15 as a myokine able to prevent intramuscular fatty infiltration, likely affecting FAPs differentiation capacities (Kang et al., 2018). In this work, the authors showed that IL-15 stimulates FAPs proliferation and it directly inhibits their adipogenic differentiation, both *in vitro* and *in vivo*, ultimately facilitating myofibers regeneration (Kang et al., 2018). Moreover, intramuscular administration of a recombinant IL-15 prevented fat accumulation in the murine model of glycerol-induced fatty degeneration (Kang et al., 2018). Accordingly, *in vitro* treatment of FAPs with IL-15 impaired their capacity to differentiate into adipocytes (Kang et al., 2018), likely through the induction of desert Hedgehog (DHH) signaling, a known repressor of FAPs adipogenesis (Kopinke et al., 2017). Although these results suggest a positive role for IL-15 in muscle regeneration, the evidence that IL-15 administration, and expression, correlates with increased collagen deposition *in vivo* after muscle damage (Kang et al., 2018), poses several unresolved issues that warrant further investigation. Indeed, whether IL-15 directly promotes FAPs differentiation into fibroblasts has not been tested yet. Furthermore, even though FAPs expansion and regenerative fibrogenesis have a positive impact on acute muscle regeneration (Heredia et al., 2013; Fiore et al., 2016), the evidence that IL-15 expression is positively correlated with the number of FAPs and collagen deposition in subjects with rotator cuff tear indicates that IL-15 might serve, instead, as a signal to sustain FAPs pathogenic fibrogenesis in chronically fibrotic muscles.

### TNF-α

Tumor necrosis factor-alpha (TNF-α) is a pleiotropic cytokine secreted rapidly upon muscle damage by infiltrating inflammatory cells and its impact on muscle regeneration is still under debate. Indeed, while pharmacological blockade of TNF-α has been associated with reduced muscle necrosis and amelioration of the histological profile of dystrophic muscles (Hodgetts et al., 2006; Huang et al., 2009; Piers et al., 2011; Ermolova et al., 2014). More recently, TNF-α has been implicated in preventing FAPs aberrant accumulation (Lemos et al., 2015; Fiore et al., 2016), suggesting that anti-TNF therapies might instead exert a pro-fibrotic effect.

During acute injury, TNF-α has been reported to promote muscle repair by activating promyogenic p38 signaling (Chen et al., 2007), thus inducing SCs differentiation (Palacios et al., 2010). Recently, it has been suggested that TNF-α regulates matrix production during acute damage, thus unveiling a crucial function for TNF-α in mediating FAPs apoptosis and clearance (Lemos et al., 2015; Fiore et al., 2016). Specifically, TNF-α was found to be predominantly expressed and produced by infiltrating monocytes that rapidly differentiate into pro-inflammatory macrophages (M1) that accumulate in close proximity to expanding FAPs. By using a mouse model unable to recruit circulating monocytes to damaged muscles [the C–C chemokine receptor type 2 (Ccr2)^–/–^ mouse strain (Warren et al., 2005)], Lemos et al. (2015) elegantly demonstrated that in the absence of infiltrating TNF-a-producing macrophages, FAPs accumulate in the sites of damage and aberrantly differentiate into fibrogenic cells. Inflammatory cell-derived TNF-α production is therefore required for FAPs clearance to prevent pathological ECM accumulation. Of note, this physiological role is altered in chronically damaged muscles, such as those of dystrophic mice, where the abundance of transforming growth factor beta 1 (TGF-β1) signaling impairs the pro-apoptotic effects of TNF-α on FAPs (Lemos et al., 2015). These data might offer a possible explanation for the apparent controversial results reporting a positive effect of anti-TNF-α therapies on dystrophic mice (Hodgetts et al., 2006; Huang et al., 2009; Piers et al., 2011; Ermolova et al., 2014). Indeed, when TGF-β1 is abundant, as in chronic degenerating muscles, the anti-fibrotic role of TNF-α is irrelevant and pharmacological approaches aimed at inhibiting its activity most likely exert their beneficial effects through targeting of the pro-myogenic, SC-mediated function of TNF-α.

### TGF-β

The transforming growth factor beta (TGF-β) superfamily comprises pleiotropic and multifunctional secreted peptides implicated in a wide range of cell functions, including tissue homeostasis and repair, immune and inflammatory responses, ECM deposition, cell differentiation, and growth (Biernacka et al., 2011; Meng et al., 2016). Studies in a wide range of experimental models have firmly established TGF-β1 as a crucial mediator of fibrinogenesis and inhibition of its activity has consistently been associated with reduced fibrosis (Biernacka et al., 2011; Meng et al., 2016).

In the context of skeletal muscle, inhibition of TGF-β1 has been linked to improvement in muscle regeneration and decreased fibrosis (Davies et al., 2016; Song et al., 2017; Zhang et al., 2019), consistent with the importance of the TGF-β1 signaling in regulating both regeneration and matrix production. Several works have elucidated the detrimental, cell-autonomous, impact of TGF-β signaling on muscle stem cells by inhibiting their activation (Carlson et al., 2008; Wang et al., 2016) and terminal differentiation (Carlson et al., 2008) while promoting a fibrogenic switch in chronically degenerating muscles (Biressi et al., 2014).

Nonetheless, recent findings point toward a prominent role for inflammatory cell-derived TGF-β signaling in the survival and fibrotic differentiation of FAPs. Specifically, during chronic muscle damage, macrophages express and secrete high levels of TGF-β1, antagonizing the TNF-mediated apoptosis of FAPs, and instead induce their fibrogenic differentiation and consequent ECM deposition (Lemos et al., 2015; Davies et al., 2016; Fiore et al., 2016; Juban et al., 2018). Thus, in conditions of chronic muscle damage, TGF-β1 acts as a dominant, pro-survival signal that overrides the beneficial effect of the pro-inflammatory cell-derived, and anti-fibrotic cytokine, TNF-α. Thus, treatment with nilotinib, via specific inhibition of TGF-β1-induced p38 signaling, restores FAPs apoptosis and prevents fibrotic accumulation in dystrophic mice (Lemos et al., 2015).

Of note, FAPs from chronic fibrotic dystrophic muscles have been identified as the main source of TGF-β-activating enzymes (Juban et al., 2018). Indeed, once released, latent TGF-β1 must be activated, either via enzymatic or mechanical mechanisms, to exert its properties and to bind to its receptors (Travis and Sheppard, 2014). FAPs exhibit high expression of a series of latent TGF-β1 activators, among which matrix metallo proteinase 14 (MMP14) and bone morphogenetic protein 1 (BMP1) are able to activate the latent TGF-β1 released by pro-inflammatory macrophages (Juban et al., 2018). Notably, pharmacological inhibition of BMP1 or MMP14 reduced muscle fibrosis in dystrophic mice resulting in increased muscle fiber size and reduced necrosis (Juban et al., 2018).

In summary, these data support a model through which chronic inflammation and fibrosis reciprocally sustain themselves in degenerating dystrophic muscles, by reinforcing a regulatory cross-talk between inflammatory cells and FAPs.

## Molecules Secreted by FAP*s*

### IL-6

In skeletal muscle, IL-6 is classified as a *myokine* produced and released by muscle fibers in response to contraction (Pedersen and Febbraio, 2008). It promotes lipolytic and anti-inflammatory beneficial effects during exercise (Pedersen et al., 2003), working as an energy sensor and exerting both local and endocrine metabolic effects.

Interleukin-6 regulates both muscle hypertrophy and regeneration (Muñoz-Cánoves et al., 2013). Indeed, IL-6 knockout mice show a reduced hypertrophic response to overloading, ascribed to impaired myonuclei incorporation as a consequence of the defective proliferation and migration of SCs. Treatment with IL-6 promotes murine SCs proliferation, via regulation of cell-cycle associated genes, Cyclin D1 and c-Myc (Serrano et al., 2008), while during regeneration the IL-6/STAT3 axis controls SCs fate (Tierney et al., 2014). Interestingly, FAPs were identified as one of the main source of IL-6 during muscle regeneration. Upon muscle injury, IL-6 expression remains constant in myogenic progenitors but increases nearly 10-fold in FAPs and *in vitro* co-culture experiments have shown that IL-6 mediates the pro-myogenic activity of FAPs (Joe et al., 2010).

The positive effect of IL-6, and others myokines, is normally associated with transient production and short-term action. By contrast, persistent inflammatory conditions, denervation, and some types of cancer and other chronic diseases have been associated with long-lasting elevated IL-6 levels. In agreement with this notion, IL-6 has been shown to promote skeletal muscle atrophy (Haddad et al., 2005). Accordingly, in denervated muscles, FAPs show a persistent activation of IL-6, thus promoting muscle atrophy without other systemic effects (Madaro et al., 2018). Notably, *in vivo* pharmacological inhibition of IL-6 effectively counteracts denervation-mediated muscle atrophy (Madaro et al., 2018) and accumulation of FAPs with hyper-activation of IL-6 signaling has also been found in mouse models of amyotrophic lateral sclerosis (ALS) (Contreras et al., 2016; Madaro et al., 2018).

Taken together, these observations suggest two apparently opposing effects of FAPs-derived IL-6 during muscle regeneration or in denervation-induced muscle wasting, and further studies are needed to shed light on the molecular mechanisms behind these apparently contradictory roles.

### IL-33

Interleukin-33 is a nuclear chromatin-associated cytokine, belonging to the IL-1 family, and constitutively expressed in the nucleus of a wide variety of cell types, including fibroblasts, epithelial cells, and endothelial cells (Carriere et al., 2007). IL-33 appears to function as an *alarmin* (alarm signal) that is rapidly released upon cellular damage and stress (Liew et al., 2016) and mediates a potent effect on the activation of regulatory T cell lymphocytes (Treg) (Matta et al., 2014; Alvarez et al., 2019).

In skeletal muscle, the major IL-33-producing cell type has been identified within the FAP cell population (Kuswanto et al., 2016). FAPs start to express IL-33 within 6–12 h after acute injury, inducing proliferation of muscle resident Treg (Kuswanto et al., 2016). As previously demonstrated, Treg cells promote muscle repair, accumulating in both acutely and chronically injured skeletal muscles (Burzyn et al., 2013; Castiglioni et al., 2015; Panduro et al., 2018). Interestingly, a severe decline in Treg accumulation, caused by an impairment in IL-33-producing FAPs, has been linked to regeneration defects in aged muscles (Kuswanto et al., 2016). On the other hand, *in vivo* treatment with IL-33 restored the Treg population in injured muscles of old mice, enhancing tissue regeneration.

Intriguingly, IL-33-expressing FAPs have been found in close association with muscle spindles (Kuswanto et al., 2016), which are stretch-sensitive mechanoreceptors that lie within the skeletal muscle and comprise both sensory and motor neurons. This finding raises the possibility that FAPs might function as mechano-cellular sensors that modulate the cross-talk between neural and immune cells to facilitate proper homeostatic reorganization of skeletal muscle and neural circuits upon injury. In agreement with this possibility, IL-33 expression is increased in fibroblasts upon mechanical stress (Kakkar et al., 2012) and PDGFRα^+^ mesenchymal precursors, found within the endoneurium of peripheral nerves, have been recently implicated in tissue repair and regeneration (Carr et al., 2019).

### WISP1

Wnt family member 1 (WNT1) inducible signaling pathway protein 1 (WISP1) is encoded by the cellular communication network factor 4 (CCN4) gene, a member of the CCN family of matricellular proteins that are involved in diverse biological processes, such as ECM remodeling, tissue repair, and tumor growth. CCN4/WISP1 is important in the musculoskeletal system, where it regulates osteogenesis and chondrogenesis, as well as skin repair (Ono et al., 2011, 2018; Maeda et al., 2015).

A recent study showed that in young mice CCN4/WISP1 is upregulated in FAPs following muscle injury, but this induction is lost in FAPs of old muscles (Lukjanenko et al., 2019). The FAP-secreted WISP1 plays an important role in SCs expansion and asymmetric commitment to myogenic differentiation. Indeed, similar to aging, the loss of WISP1 in knockout mice affects SCs function and impairs myogenesis. In agreement with this, the transplantation of young, but not aged or WISP^–/–^ FAPs, rescues the myogenic dysfunction of aged SCs and their regeneration ability (Lukjanenko et al., 2019). Even better, systemic treatment with recombinant WISP1 mimics rejuvenation beneficial effects, opening new prospects in the use of this approach as a strategy to counteract aging and associated muscular diseases.

Interestingly, these findings together with the reported impaired interplay between FAPs and Treg during aging (Kuswanto et al., 2016), the sensitivity of FAPs to muscle denervation (Madaro et al., 2018), and the recently reported atrophic phenotype of FAPs-depleted skeletal muscles (Wosczyna et al., 2019) clearly point toward FAPs as promising new cellular targets for the treatment of muscle defects associated with sarcopenia.

### Follistatin

Follistatin is a potent natural antagonist of myostatin and activin A, two TGF-β superfamily cytokines implicated in muscle growth inhibition, and it is therefore a potent pro-myogenic factor (Lee, 2007; Nakatani et al., 2008; Guo et al., 2009; Kota et al., 2009; Rodino-Klapac et al., 2009; Winbanks et al., 2012).

In the context of skeletal muscle regeneration, follistatin expression is induced 12 h after muscle injury (Iezzi et al., 2004) and remains elevated for 5 days, concurrent with SCs activation. Of note, FAPs have been described as the major source of follistatin, displaying 10-fold higher expression levels than SCs (Mozzetta et al., 2013; Formicola et al., 2019). Follistatin is considered the central mediator of the fusogenic effects exerted by histone deacetylase inhibitors (HDACi) on skeletal muscles (Iezzi et al., 2004; Minetti et al., 2006; Mozzetta et al., 2013). Indeed, HDACi treatment in dystrophic mice induces the upregulation of follistatin in muscle progenitor cells, promoting the formation of multinucleated myotubes. In agreement with its pro-myogenic activity, follistatin knock-down in FAPs reduced the ability of HDACi to stimulate SCs-mediated formation of myotubes, suggesting a crucial role of FAP-derived follistatin as a mediator of the pro-differentiative activity of FAPs (Mozzetta et al., 2013).

A proper balance between follistatin and its antagonists is crucial to preserve reciprocal functional interactions between FAPs and SCs and to preserve muscle homeostasis (Baccam et al., 2019; Formicola et al., 2019). Indeed, pharmacological inhibition of the activin receptor type-2B pathway (AcvR2B), which blocks both myostatin and activin A activity, reverses muscle atrophy in SC-depleted skeletal muscles, while also restoring stem cells regenerative potential (Formicola et al., 2019). Of note, beneficial effects of AcvR2B targeting in SCs-depleted muscles are accompanied by an increased number of FAPs (Formicola et al., 2019), an observation that further supports the notion that restoration of a proper balance of regulatory factors between the different cells within the regenerative niche is key for muscle repair.

Once again, a role of FAPs emerges not only in regulating muscle regeneration but also in mediating signaling pathways associated with maintaining muscle mass. Future experiments should elucidate the possible use of FAPs as a source of trophic factors.

### IL-10

Interleukin is a broadly expressed anti-inflammatory cytokine that inhibits the activation of the innate immune system and Th1 activation, preventing inflammatory and autoimmune pathologies (Saraiva and O’Garra, 2010; Ouyang et al., 2011). Like IL-6, IL-10 is also considered a *myokine* expressed in skeletal muscle in a wide range of conditions. It influences different aspects of muscle biology, such as regeneration, exercise, metabolism, and aging (Furmanczyk and Quinn, 2003; Nunes et al., 2008; Villalta et al., 2011; Deng et al., 2012; Dagdeviren et al., 2016, 2017). Its anti-inflammatory activity has been investigated in different muscle-related disorders (Hong et al., 2009; Nitahara-Kasahara et al., 2014; Villalta et al., 2014; Dagdeviren et al., 2016, 2017; Zhang et al., 2018). The main source of IL-10 in regenerating skeletal muscle is macrophages and Tregs (Villalta et al., 2011, 2014; Deng et al., 2012) although it has also been demonstrated that FAPs increase IL-10 expression upon muscle damage (Lemos et al., 2012). This evidence is in line with the crucial pro-myogenic activity of FAPs, which likely contribute to muscle repair also through the secretion of an anti-inflammatory cytokine, such as IL-10, to counteract the anti-myogenic activity of TNF-α. Although the function of IL-10 released by FAPs has not yet been demonstrated *in vivo*, this work underscores the complexity of the interplay between inflammatory cells and the other players in muscle regeneration. Future studies are needed to better understand this mechanism.

## Concluding Remarks

**FIGURE 1:**
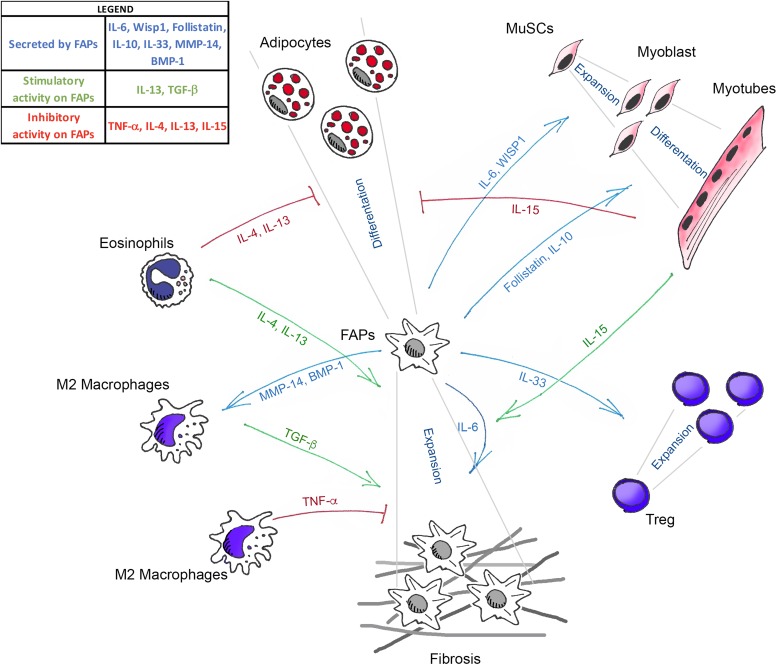
Schematic illustration showing the known mediators that govern the interaction between FAPs, muscle stem cells (MuSCs), and the different immune cells in skeletal muscle homeostasis. Green arrows (TGF-β, IL-13, IL-4, and IL-15) indicate the molecules that positively regulate FAPs expansion. Blue arrows (IL-33, IL-6, Follistatin, IL-10, WISP1, MMP-14, and BMP-1) represent the molecules secreted by FAPs that act on the different cell targets. Red lines (TNF-α, IL-4, IL-13, and IL-15) show the factors that inhibit the fibro-adipogenic differentiation of FAPs.

In conclusion, the available evidence reviewed above clearly indicates that FAPs act as crucial regulators of skeletal muscle homeostasis (Figure 1). However, several critical issues need to be addressed before defining them as the co-star of skeletal muscle repair. First, their molecular heterogeneity makes it difficult to target them genetically, to uniquely assess their requirement, and to define the function of FAP-specific expression of the different factors described above. Future single-cell transcriptomic approaches will help identify sub-populations differently altered during the diverse stages of muscle regeneration and, more importantly, in pathological situations.

Finally, the evidence of the association of FAPs with nerve structures (Kuswanto et al., 2016), and the ability of FAPs to respond to nerve lesions (Contreras et al., 2016; Madaro et al., 2018), suggest a mechano-sensitivity of FAPs and emphasize the urgency to improve our understanding of the molecular regulation governing FAPs activity during muscle adaptation.

## Author Contributions

LM and CM wrote and edited the manuscript. BB and DP wrote the manuscript.

## Conflict of Interest Statement

The authors declare that the research was conducted in the absence of any commercial or financial relationships that could be construed as a potential conflict of interest.

The handling Editor declared a shared affiliation, though no other collaboration, with several of the authors, BB, CM, and DP, at the time of review.
